# A highly efficient regeneration, genetic transformation system and induction of targeted mutations using CRISPR/Cas9 in *Lycium ruthenicum*

**DOI:** 10.1186/s13007-021-00774-x

**Published:** 2021-07-03

**Authors:** Wang Wang, Jiangmiao Liu, Hai Wang, Tong Li, Huien Zhao

**Affiliations:** Beijing Key Laboratory of Ornamental Plants Germplasm Innovation & Molecular Breeding; National Engineering Research Center for Floriculture; Beijing Laboratory of Urban and Rural Ecological Environment; Key Laboratory of Genetics and Breeding in Forest Trees and Ornamental Plants of Ministry of Education; College of Landscape Architecture, Beijing Forestry University, Beijing, 100083 China

**Keywords:** Black wolfberry, CRISPR/Cas9, Gene editing, *fw2.2*

## Abstract

**Background:**

CRISPR/Cas9 is a rapidly developing genome editing technology in various biological systems due to its efficiency, portability, simplicity and versatility. This editing technology has been successfully applied in in several important plants of Solanaceae such as tomato, tobacco, potato, petunia and groundcherry. Wolfberry ranked the sixth among solanaceous crops of outstanding importance in China following potato, tomato, eggplant, pepper and tobacco. To date, there has been no report on CRISPR/Cas9 technology to improve *Lycium ruthenicum* due to the unknown genome sequencing and the lack of efficient regeneration and genetic transformation systems.

**Results:**

In this study, we have established an efficientregeneration and genetic transformation system of *Lycium ruthenicum.* We have used this system to validate target sites for *fw2.2*, a major fruit weight quantitative trait locus first identified from tomato and accounted for 30% of the variation in fruit size. In our experiments, the editing efficiency was very high, with 95.45% of the transgenic lines containing mutations in the *fw2.2* target site. We obtained transgenic wolfberry plants containing four homozygous mutations and nine biallelic mutations in the *fw2.2* gene.

**Conclusions:**

These results suggest that CRISPR-based gene editing is effective for the improvement of black wolfberry traits, and we expect this approach to be routinely applied to this important economic fruit.

**Supplementary Information:**

The online version contains supplementary material available at 10.1186/s13007-021-00774-x.

## Background

Wolfberry is used as a traditional medicine in China and other Asian countries owing to the abundance of anthocyanins, trace minerals, vitamins and polysaccharides in the fruit [1, 2]. The wolfberry planting area is usually at about 410,000 tons 160,000 hm^2^ with 18.7 billion RMB profit in China in 2018. Black wolfberry has higher nutrient and medical values, it is more resistant to drought and saline-alkaline compared with other goji species, and the plant can be survived in the areas with 50 mm annual precipitation, as a result, the artificial cultivation of the undomesticated black wolfberry has been greatly developed in North West China, which is also labor-intensive industry due to the fruit is mostly hand harvested. Small fruit and dense sharp thorn make it more difficult to harvest. High quality of fruit such asthe large fruit size is even more important than total yield for attaining market competitiveness. As a producer, obtaining new large-fruited cultivars is a major breeding goal for grower profitability. Therefore, understanding the genetic background that controls fruit size is significant in order to improve the breeding efficiency of large-fruit varieties, which will also greatly promote the use of small-fruit wild germplasm, as it will reduce the number of generations required to obtain the commercial fruit size needed for a new cultivar. The genetic basis of variation in fruit size has been studied most widely in tomato to date (*Solanum lycopersicum* L.) [3, 4]. *fw2.2* was the first fruit weight gene identifified from tomato as a result of domestication, it is a major quantitative trait locus that regulates fruit size and weight, and natural genetic variation at this locus alone can change the size of fruit by up to 30% in tomato between large, domesticated tomatoes (*Lycopersicon esculentum* Mill.) and their small-fruited wild relatives [5].The “small-fruit” alleles at the *fw2.2* locus exists in all wild tomatoes, whereas all cultivated tomatoes tested are fixed for “large-fruit” alleles [6]. *fw2.2* were negatively correlated with cell division, that is, the higher expression level, the lower cell number, therefore affecting the mitotic activity during early fruit development [5, 7]. Many studies demonstrate the role of cell number in determining fruit size in fruit crops such as peach [8], sweet cherry [9], olive [10], and apple [11]. Due to *fw2.2* homologous gene provides an excellent source of candidates for fruit size regulation in other domesticated species [12, 13], therefore, *fw2.2* was further studied by CRISPR/Cas9-mediated gene editing technology in *Lycium*.

The CRISPR/Cas9 system has been utilized for genome engineering in several important plants of *Solanaceae*, including tobacco, tomato, potato, petunia and groundcherry [14, 15]. Among horticultural crops, tomato has been receiving increasing research attention regarding genome editing compared with other crops: 42% of genome-editing studies are related to tomato, whereas 13% of which are related to potato [15]. CRISPR/Cas9-mediated knock-out of polyphenol oxidase genes in eggplant has also been reported [16]. However, this powerful technology for genome editing has not yet been used in *Lycium*.

In this study, we have established an efficient regeneration and genetic transformation system for black wolfberry, and we first adapted a schematic workflow and cloning strategy for black wolfberry CRISPR/Cas9 system in order to breeding elite cultivars with bigger fruits, since the stem is covered with thorns and the fruit is more difficult to harvest. The results show that this CRISPR/Cas9 system is effective in *Lycium ruthenicum*.

## Results

### Structure and phylogenetic analysis of *fw2.2* gene of *Lycium ruthenicum*

**Fig. 1:**
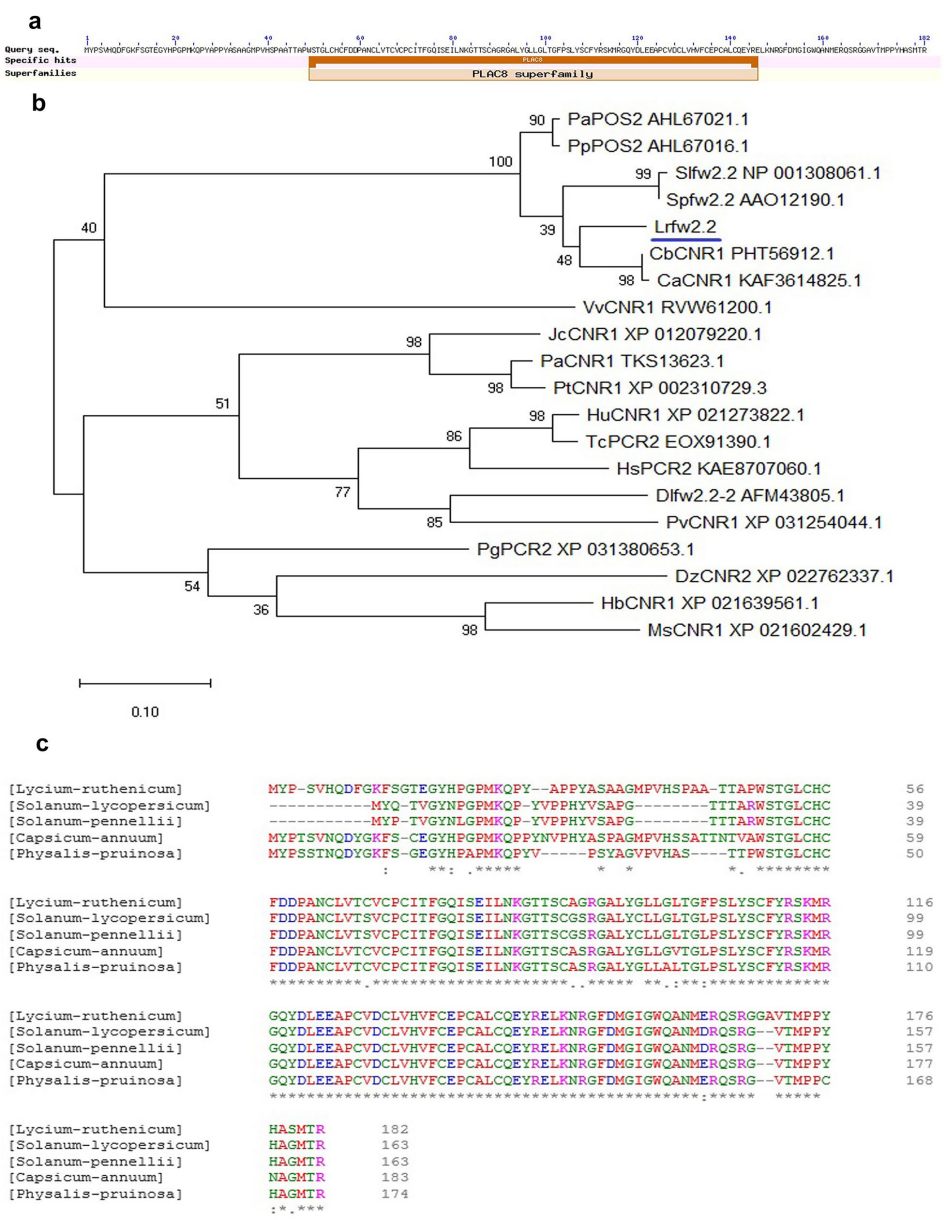
Conserved domain and phylogenetic analysis of *Lycium ruthenicum fw2.2*. **a** Conserved domain analysis of *fw2.2.* **b** Phylogenetic tree of the predicted CNR/FWL proteins, the plants corresponding to the CNR/FWL proteins are: PpPOS2, *Physalis pruinosa*; PaPOS2, *Physalis aequata*; Slfw2.2, *Solanum lycopersicum*; Spfw2.2, *Solanum pimpinellifolium*; CaCNR1, *Capsicum annuum*; CbCNR1, *Capsicum Baccatum*; VvCNR1, *Vitis vinifera*; JcCNR1, *Jatropha curcas*; PaCNR1, *Populus Alba*; PtCNR1, *Populus trichocarpa*; HuCNR1, *Herrania umbratica;* TcPCR2, *Theobroma cacao*; HsPCR2, *Hibiscus syriacus*; Dlfw2.2–2, *Dimocarpus longan*; PvCNR1, *Pistacia vera*; PgPCR2, *Punica granatum*; DzCNR2, *Durio zibethinus*; HbCNR1, *Hevea brasiliensis*; MsCNR1, *Manihot esculenta*. **c** Multiple sequence alignment of *fw2.2* gene of *Lycium ruthenicum* and other species in Solanaceae, the gene accession number are: *Solanum lycopersicum*: NP_001308061.1; *Solanum pennellii*: AAO12196.1; *Capsicum annuum*: PHT91598.1; *Physalis pruinosa*: AHL67016.1

The full-length DNA of *Lycium ruthenicum fw2.2* is 2323 bp, it is divided into three exon regions by introns, the lengths of which are exon 1:259 bp, exon 2:210 bp, exon 3:80 bp. The length of the cDNA sequence is 549 bp, encoding 182 amino acids. This black wolfberry gene containing a PLAC8 (Placenta-specific 8) conserved domain belongs to the *fw2.2* family (Fig. 1a). We used MEGA-X to re-align the homologous protein sequences found by BLASTp alignment on NCBI. The results showed most of these proteins belonged to *FWL*/*CNR* family, and few of them are PCR (Plant cadmium resistance) family proteins (Fig. 1b), both of which have PLAC8conserved domain and similar structures. The selected Solanaceae fw2.2 homologous protein including *Lycium ruthenicum* fw2.2, Lycopersicon fw2.2, Physalis POS2, and Capsicum CNR1 clustered together with more than 85% amino acid sequence similarity (Fig. 1c), indicating that fw2.2 protein is relatively conserved in Solanaceae. *FW2.2*-like genes have been renamed as the Cell Number Regulator (CNR) family, which has a negative regulatory effect on cell number [17, 18]. Studies have shown that *fw2.2* can account for 30% and 47% of the fruit size phenotypic variation in *Lycopersicon pimpinellifolium* and *Lycopersicon pennellii*, respectively [19]. The *POS2* (physalis organ size 2) in *Physalis floridana* encodes a putative ortholog of *fw2.2*, which can regulate the cell cycle and has a negative effect on fruit size [20]. The function of CNR1 in pepper is unknown. Both tomato *fw2.2* and physalis *POS2* negatively regulate cell division and affect fruit size. Therefore, we infer that the black wolfberry *fw2.2* gene, which has a close phylogenetic relationship with them, may also have similar functions.

### Establishment of *Lycium ruthenicum* regeneration system

The seeds of *Lycium ruthenicum* were disinfected and inoculated on 1/2 MS medium, we have established a suitable regeneration system of *Lycium ruthenicum* after the induction and differentiation of leaf-derived callus, and the rooting of regenerated shoots (Additional file 1: Table S1). After 15 days of callus induction, different media with different hormonal combinations had a high callus induction rate (100%) but different callus growth status. Among them, callus grows best on A3 medium (MS + 0.5 mg/L 6-BA + 0.5 mg/L NAA) with green appearance, loose structure, no browning, and low vitrification, which is the most suitable medium (Additional file 1: Table S1). The callus was subsequently transferred to B1–B10 medium to induce differentiation, and the differentiation rate and multiplication coefficient were counted after 30 days. We found that the callus was not differentiated or the differentiation rate was low without 6-BA (B5) or the concentrations of 6-BA were higher (B1-B4). Both the differentiation rate and multiplication coefficient were significantly increased on the medium supplemented with low concentration of 6-BA (less than 0.5 mg/L), which reached their highestwhen the callus was cultured on shoot induction medium (B7:MS + 0.2 mg/L 6-BA + 0.05 mg/L NAA). The callus did not turn brown and showed a relatively low degree of vitrification (Additional file 1: Table S1). The differentiated shoots were separated and transferred on 1/2 MS medium without hormones, and rooting can be induced after 15 days.

### Target site selection and sgRNA Design

The CRISPR online design tool [21]was used to find common and unique CRISPR single guide RNA targets in a set of similar sequences for the reference tomato genome( *Solanum lycopersicum* genome, SL2.40)Based on the selection criteria that the PAM site sequence is NGG, the GC content is 40%–70%, the target sequence avoids spanning intron regions and the occurrence of more than 4 consecutive T bases, the top sgRNA were selected for the knockout experiments. The sgRNA used in this study are shown in Table S2. The position of fw2.2–1 and fw2.2–2 is 1857–1879 and 171–193 respectively, spanning 1664 bases.

### Establishment of *Lycium ruthenicum* genetic transformation system and efficiency evaluation of CRISPR/Cas

**Fig. 2:**
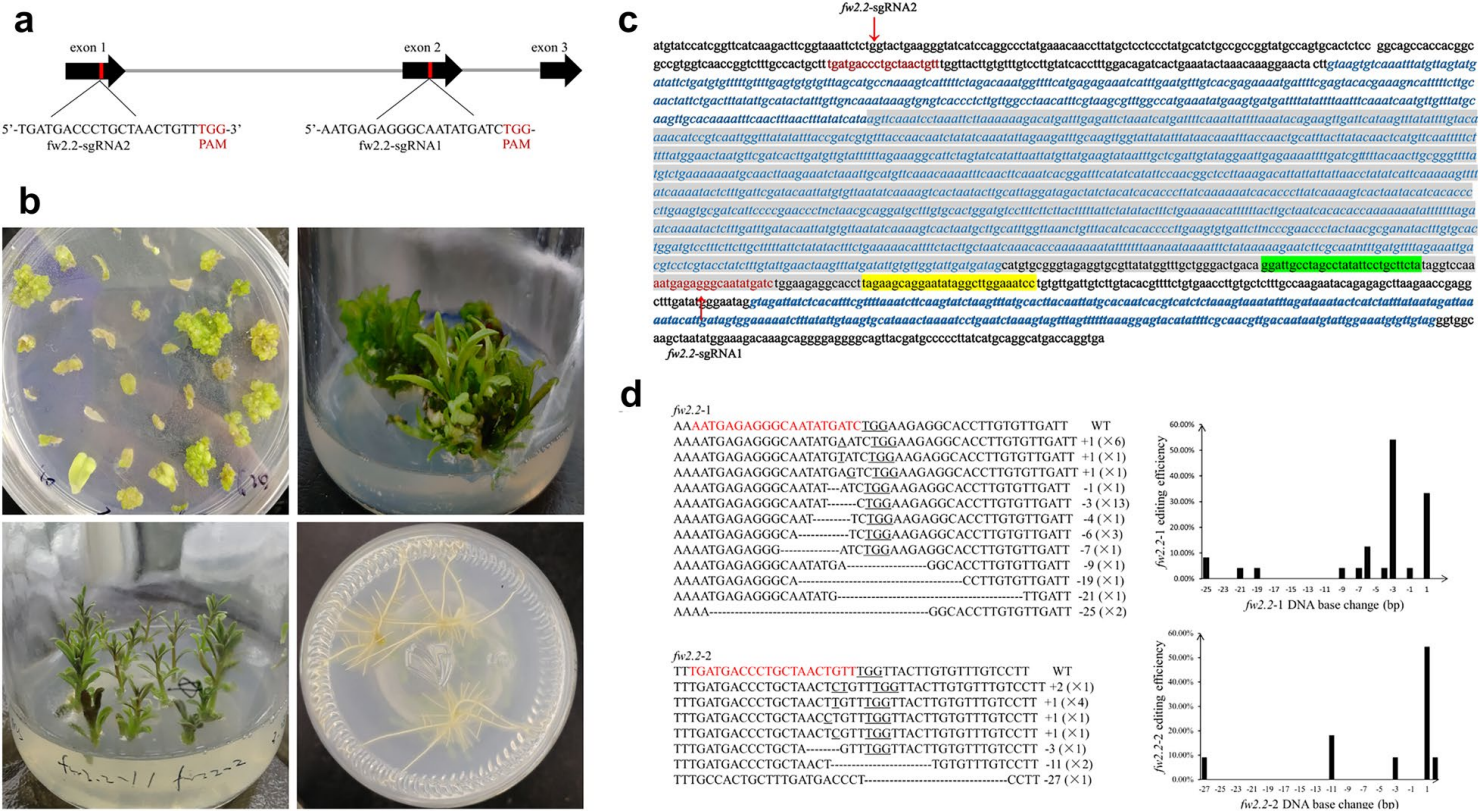
Genome editing in *Lycium ruthenicum* using CRISPR/Cas9 technology. **a** *fw2.2* gene structure and sequences of the target sites. Black boxes: exons; grey lines: introns; sgRNA target sites and the PAM regions (Red). **b** The process of transformation (Two-week-old callus; differential shoots after four-week of subculture; elongated shoots after six-week of subculture; eight-week-old rooted transformant). **c** The combination of large fragment deletions with insertions in a T0 plant. The 1515 bp in italic is the intron. 1281 bp deletions are labeled in gray. 29 bp insertions shown in yellow are almost the inversion of the fragment nearby labeling with green. **d** Editing type and preference of different target sites

Two single sgRNA (sgRNA1 and sgRNA2) and dual sgRNAs of *fw2.2* were designed that target different sites after the whole sequence being cloned and phylogenetic analyzed. The target site of *fw2.2*-sgRNA1 and *fw2.2*-sgRNA2 are located in exon 2 and exon 1 respectively (Fig. 2a). The high efficient Agrobacterium tumefaciens-mediated transformation of black wolfberry was performed here using the leaves under the conditions of 0.2 Agrobacterium concentration (OD600), 10 min of infection, 200 μmol/L acetosyringone supplement and 2 days of co-cultivation (Additional file 1: Table S3). The 40 mg/L hygromycin selective pressure plus 200 mg/L carbenicillin were used to select resistant plants and inhibit Agrobacterium growth. The entire experimental cycle took approximately 2 months from incubation to mutant identification with 2 days of co-cultivation, 15 days of callus production, 30 days of differentiation and sub-culture (Fig. 2b). The details of the transformation media can be found in Table 1. The mean transformation efficiencies of these three lines were 2.66%, 1.18% and 5.33%, respectively.

**Table 1:** The media information used in the transformation

Medium name	Medium composition
Co-culture medium	MS + 0.5 mg/l 6-BA + 0.5 mg/l NAA
Resistant callus selection medium	MS + 0.5 mg/l 6-BA + 0.5 mg/l NAA + 40 mg/l Hyg + 200 mg/l Carb
Resistant bud selection medium	MS + 0.2 mg/l 6-BA + 0.05 mg/l NAA + 40 mg/l Hyg + 200 mg/l Carb
Rooting medium	1/2 MS + 200 mg/l Carb

In this study, a lot of resistant seedlings were produced. Twenty-one out of twenty-two, six out of eleven and fifteen out of sixteen bigger plants were detected with novel mutations in the sgRNA1, sgRNA2 and sgRNA1/sgRNA2 regions. As shown in Table 2, the gene editing efficiency of *fw2.2*–1 target is high (95.45%), while that of *fw2.2*–2 target is low (54.55%). However, the editing efficiency of homozygous mutants (18.18%) and biallelic mutants (9.09%) is higher than those of *fw2.2*–1 (4.55%).

**Table 2:** The editing efficiency and mutation type in single-target system and dual-target system

Vector name	Editing efficiency	Heterozygous rate (%)	Homozygous rate (%)	Double allele rate (%)
1300cas9-fw2.2-sgRNA1	95.45	86.36	4.55	4.55
1300cas9-fw2.2-sgRNA2	54.55	27.27	18.18	9.09
*fw2.2*-sgRNA1 target site	87.5	31.25	12.50	43.75
*fw2.2*-sgRNA2 target site	43.75	43.75	0.00	0.00

Both 1300cas9-*fw2.2*-sgRNA1 and 1300cas9-*fw2.2*-sgRNA2 are single-target vectors; *fw2.2*-sgRNA1 and *fw2.2*-sgRNA2 are two different target sites in the dual-target vector

The dual-sgRNA CRISPR/Cas9 system was highly reproducible and highly efficient since it could result in more reliable loss-of-function alleles that lack a large essential part of the gene [22]_._ Here we found the editing efficiency of *fw2.2* in the homozygote/biallelic mutations altogether (56%) by the dual-sgRNA CRISPR/Cas9 system is more than twice (27%) of that by the sgRNA CRISPR/Cas9 system, though the editing efficiency of the dual-sgRNA system is only 93.75%, which is a little less than that of (95.45%) the sgRNA1 system (Tables 2 and 3). It was also found that there was a 1281 bp segment deleted and 29 bp insertion in the *fw2.2* of a T0 plant (Fig. 2c), which is similar to the result such as 934-bp deletion mutation at the AtMIR169a locus of *Arabidopsis *[22].

**Table 3:** The editing efficiency in dual target system

Vector name	Editing efficiency	Single target editing efficiency	Double target editing efficiency
1300cas9-*fw2.2*–1/2	93.75	56.25	37.5

### Expression analysis of *fw2.2* in gene-edited seedlings by quantitative real-time PCR

**Fig. 3:**
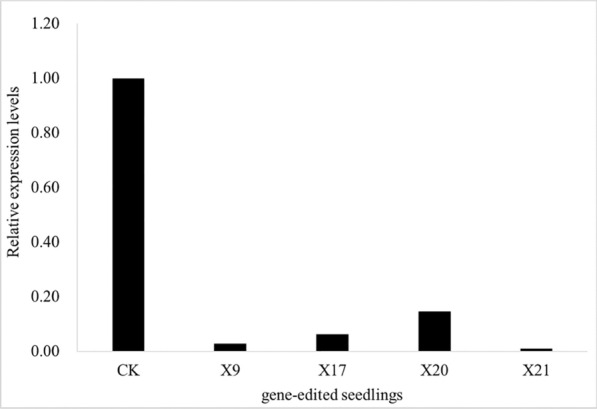
Verification of the relative expression of *fw2.2* in gene-edited seedlings

We found that the expression level of the *fw2.2* gene in various gene-edited seedlings was significantly lower than that in wild plant (Fig. 3), and the lowest was 0.01 in X21, which had a large fragment sequence deletion of 1281 bp. X9 is a homozygous mutant, the expression level of *fw2.2* gene in which was only 0.03, and followed by X17 and X20, which were 0.06 and 0.15 respectively. These data suggest that CRISPR/Cas9 had a significant effect on *fw2.2* expression. When a gene expression cassette is introduced into a genome by CRISPR/Cas9, the sgRNA target gene becomes inactive because of disruption of the gene, which probably influences the gene expression and the developmental characteristics of the modified strain if the inactive gene is generally involved in metabolism.

## Discussion

Creating crops with various traits is challenging to conventional breeding since it is time- consuming and labor-intensive. Recent advances in gene editing technology open the exciting prospect of creating novel crops via de novo domestication, the application of this technology speeds up the process of obtaining plant excellent traits and greatly improves the accuracy [23, 24]. Until now, successful genome editing technology mediated by CRISPR/Cas9 was demonstrated in Solanaceae, including tomato [25], potato [26], tobacco [27], Petunia [28]*,* Physalis [14] and eggplant [29], fully demonstrating the broad prospects of molecular genetic technology for crop improvement. However, due to the complex genomes, slow growth cycles, difficulties of transformation and a lack of genomic information, it remains a long and arduous task to apply CRISPR/Cas9 to *Lycium ruthenicum*. In this study, we established a suitable regeneration and genetic transformation system for *Lycium ruthenicum*, which laid the foundation for the subsequent successful transformation of CRISPR/Cas9 plasmids. Studies have shown that simultaneous targeting of multiple regions of the same gene or functionally‐redundant genes can improve gene editing efficiency [30]. We constructed a dual-target editing vector pCAMBIA1300-*fw2.2*–1/2 as well as a single-target editing vectors pCAMBIA1300-*fw2.2*–1 and pCAMBIA1300-*fw2.2*–2. And both target sites were optimized with CRISPR online design tool using the tomato reference genome to maximize activity and minimize off-target effects of CRISPR-Cas9. Different mutants were detected in all tested targets in the T0 transgenic black wolfberry plants, including homozygous, biallelic and heterozygous. The majority of the detected mutations were 3 base deletion occurring at 3–5 bp upstream of PAM site (Fig. 2d).The deletion of the 1281 bp large fragment and 29 bp insertion in the *fw2.2* (Fig. 2c) were also found in the T0 plant. All these results demonstrate that the CRISPR/Cas9 system is an efficient tool for generating target mutations in black wolfberry plants.

A recent report has demonstrated *fw2.2* which encodes a negative cell number regulator increases fruit weight by approximately 30% between the domesticated tomato and its wild relatives in the genus,and *fw2.2* was selected as domestication gene to increase tomato fruit size via de novo domestication [5]. *FW2.2* belongs to a multigene family named the *CELL NUMBER REGULATOR* (*CNR*) family. More and more studies have shown that the *CNR*/*FWL* family plays an important regulatory role in increasing fruit size in several fruit species, including papaya [31], peach [32], cucumber [33], grapevine [34], cherry [9], eggplant [35], tomato [5], avocado [12], pear [36], *Physalis floridana *[20]. Increased transcript level of *CNR*/*FWL* is negatively correlated to fruit size, further indicating that *CNR*/*FWL* is highly conserved in different species. *CNR*/*FWL* also regulates organ size in cereal and leguminous species, such as in maize [37], rice [17], soybean [38], which is achieved by regulating the number of cells. In our study, the *fw2.2* gene of *Lycium ruthenicum* that we identified has high homology with other species, studies have shown that specific *fw2.2*-like proteins might share a similar biological function with the tomato *fw2.2 *[37, 38], which suggest that *fw2.2* in *Lycium ruthenicum* might be associated to regulation and/or repression of fruit cell division. Whether we could produce the bigger fruit phenotypes of black wolfberry or not needs to wait until next summer.

## Conclusions

This study is the first to use CRISPR/Cas9 technology for targeted genome editing in *Lycium ruthenicum*, and demonstrates that the system has high editing efficiency. The results obtained in this study can help to understand the mechanism of fruit development. Meanwhile, the regeneration and genetic transformation system established in this study can provide theoretical guidance for transgenic research of *Lycium ruthenicum*. The targeted knockout of desired genes using CRISPR/Cas9 is also of great significance for developing new traits of *Lycium ruthenicum*. This result provided a suitable method for de novo domestication of wild black wolfberry though the effect of successful editing on fruit development needs further morphological analysis and transcriptome sequencing. We expect this approach will be routinely applied to this important economic fruit in the near future.

## Methods

### Plant materials

Wild *Lycium ruthenicum* seeds were obtained from Alxa, Inner Mongolia, and rinsed under running water for 2 h, then disinfected with 75% alcohol for 30 s, rinsed with sterile water once, then rinsed with 4% sodium hypochlorite for 3 min, the seeds were rinsed with sterile water 3 times at last and then inoculated in 1/2MS medium, placed at 25 °C and 14 h/d light (1500 ~ 2000 lx) in the tissue culture room to obtain tissue culture seedlings for later gene editing study.

### *fw2.2* gene cloning and sequence analysis

The *fw2.2* sequences of *Solanum lycopersicum* (NM_001321132.1), *Lycopersicon pennellii* (AY097189.1), *Physalis coztomatl* (KJ155739.1), *Physalis lanceifolia* (KJ155740.1), *Physalis pruinosa* (KJ155742.1), *Physalis peruviana* (KJ155745.1), *Physalis ixocarpa* (KJ155746.1), *Physalis mexicana* (KJ155748.1) were applied as seed sequences to search the *Lycium ruthenicum* transcriptome sequencing database (SRA:SSR7700825), and a highly homologous sequence was obtained based on BLAST search. The Pfam database [39] and the Conserved Domain Database (CDD) of the NCBI [40] were used to analyze the obtained sequence that contained the known conserved domains and motifs. The total RNA and genomic DNA of *Lycium ruthenicum* were extracted for PCR assay by gene-specific primers to amplify the full-length sequence of *fw2.2 *(Additional file 1: Table S4). The exon and intron regions were analyzed by alignment of the compiled sequence with corresponding cDNA and the canonical GT/AG rule. The sequences of *fw2.2* proteins producing significant alignments were searched on BLASTP programs to construct phylogenetic tree, and the neighbor-joining (NJ) method of the MEGA-X was used to analyze phylogenetic relationships, the confidence limits of each branch in the phylogenetic tree were assessed by 1000 bootstrap replications and expressed as percentage values.

### CRISPR/Cas9 vector construction

**Fig. 4:**
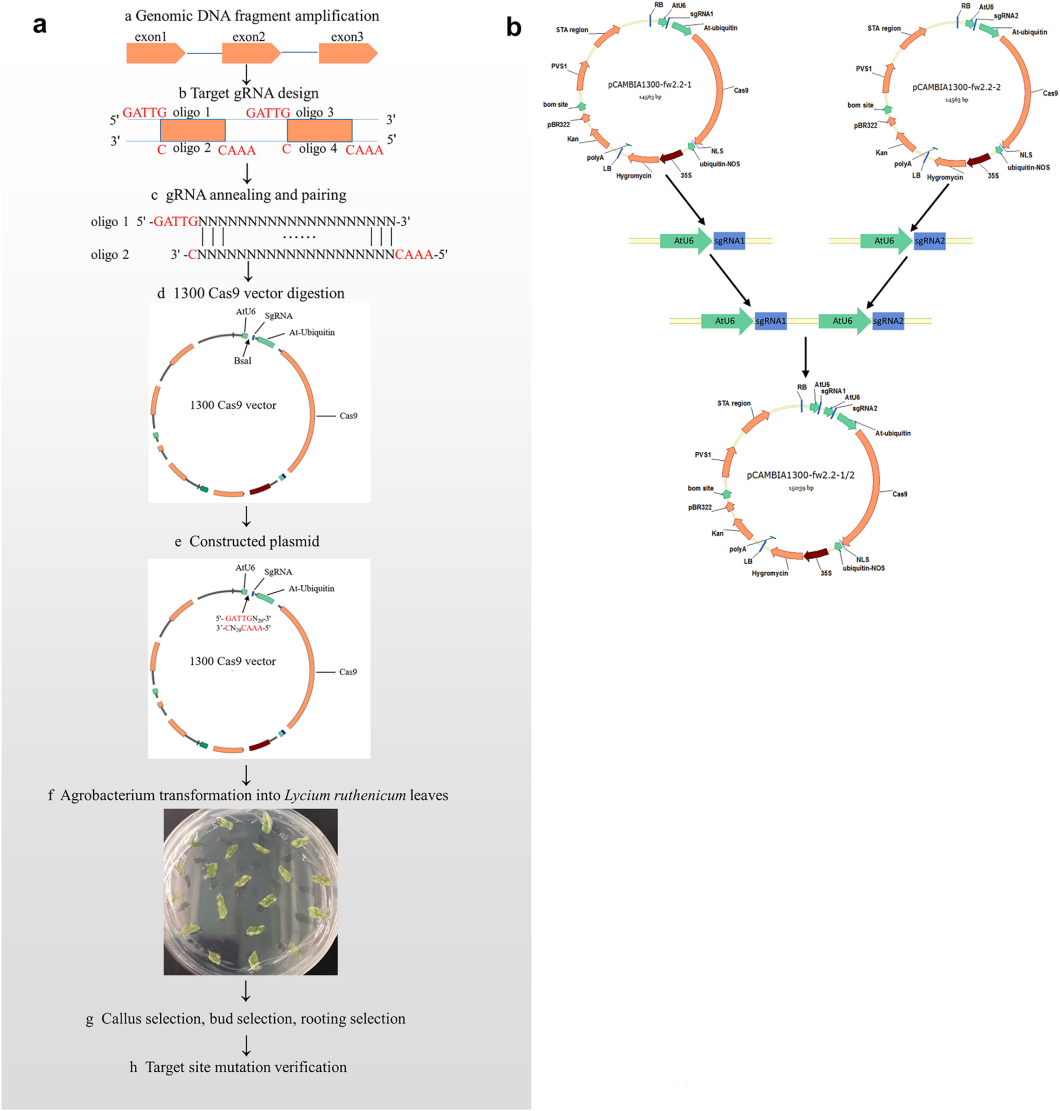
Construction of single target vector and dual target vector. **a** Single-target vector construction. **b** Dual- target vector construction

The CRISPR online design tool was used to analyze the target location and GC content, etc., and the reference genome of *Solanum lycopersicum*(SL2.40) was used to assess the target specificity (Additional file 1: Table S2). The single- target vector construction method was shown in Fig. 4a, the primers *fw2.2–*1-sgRNA and *fw2.2–*2-sgRNA with target adapter (Additional file 1: Table S5)were used to anneal and pair following the procedure (20 μl reaction system contains 1 μl forward primer, 1 μl reverse primer and 18 μl anneal buffer, under the condition of 95 ℃ 1 min, 0.1 ℃/s cooling down to 25℃). The pCAMBIA1300-sgRNA/Cas9 vector was digested with BsaI (NEB) under 37 ℃for 2 h (25 μl reaction system contains 2 μg plasmid vector, 0.5 μl BsaI, 2.5 μl 10 × Cutsmart Buffer and ddH2O). The double-stranded primer was then ligated with the digested vector to obtain single-target editing vector pCAMBIA1300-*fw2.2*–1 and pCAMBIA1300-*fw2.2*–2.

The dual-target vector construction method was shown in Fig. 4b. The above-obtained pCAMBIA1300-*fw2.2*–1 and pCAMBIA1300-*fw2.2*–2 were used as templates, the primers containing EcoRI restriction site were used for PCR amplification to obtain *fw2.2*-AtU6-sgRNA1 and *fw2.2*-AtU6 -sgRNA2 (Additional file 1: Table S6). The two sgRNA expression cassettes were respectively digested with EcoRI and then ligated with T4 ligase to obtain the two-site expression cassette *fw2.2*-AtU6-sgRNA1-AtU6-sgRNA2. Both the *fw2.2*-AtU6-sgRNA1-AtU6-sgRNA2 and pCAMBIA1300-sgRNA/Cas9 vector were digested with HindIII and XmaI, respectively, and then ligated with T4 ligase to obtain pCAMBIA1300-*fw2. 2*–1/2 dual-site expression vector.

### Optimization of regeneration system and Agrobacterium-mediated transformation of *Lycium ruthenicum*

The leaves of the *Lycium ruthenicum* tissue culture seedlings were cut into about 0.5 cm and then transferred to MS medium (A1-A12) supplemented with various concentrations and combinations of 6-BA, NAA and 2, 4-D to induce callus (Additional file 1: Table S1). The callus induction rate was counted after 30 days based on three experiments using 30–40 explants in each treatment. The callus was subsequently transferred to MS medium (B1-B10) supplemented with various concentrations of 6-BA and NAA to induce differentiation (Additional file 1: Table S1). 30–40 explants were used in each treatment of three experiments, and explants were subcultured at intervals of 20 days. The differentiation rate and multiplication coefficient were counted and recorded after 60 days. The optimal regeneration system of *Lycium ruthenicum* was determined based on the above callus induction and differentiation experiments.

The leaves of *Lycium ruthenicum* tissue culture seedlings were used for Agrobacterium transformation. The influence of hygromycin concentration, infection time, co-cultivation time, bacterial solution concentration and acetosyringone concentration were comprehensively analyzed to explore the optimal genetic transformation conditions (Additional file 1: Table S3). The Agrobacterium cells were collected and resuspended in MS liquid medium after expanded cultivation in LB medium containing 50 mg/L Kan, 50 mg/L Gen and 50 mg/L Rif. The Agrobacterium-infected leaves were transferred to co-culture medium (MS + 0.5 mg/L6-BA + 0.5 mg/LNAA) for two days in the dark at 25℃, and then transferred to resistant callus selection medium (MS + 0.5 mg/L6-BA + 0.5 mg/LNAA + 40 mg/L Hyg + 200 mg/L Carb). After that, the resistant callus were transferred to the medium (MS + 0.2 mg/L6- BA + 0.05 mg/LNAA + 40 mg/L Hyg + 200 mg/L Carb) to obtain resistant buds. With 2 rounds of selection and culture, the resistant buds were cut and transferred to rooting medium (1/2 MS + 200 mg/L Carb) to obtain the gene editing tissue culture seedlings of *Lycium ruthenicum*.

### Identification of gene-edited *Lycium ruthenicum*

**Fig. 5:**
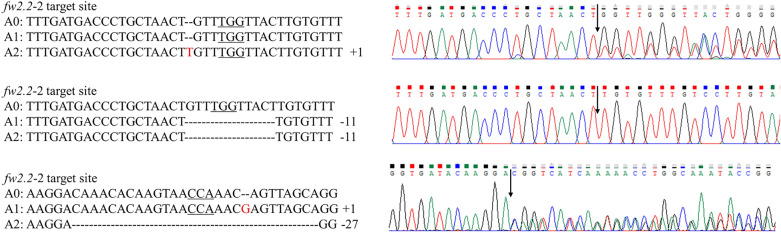
Mutation types and sequencing chromatograms of gene-edited seedlings

Genomic DNA was extracted from the wild-type (Wt) and resistant seedlings for PCR assay by primers spanning the upstream and downstream gRNA target sites to detect CRISPR/Cas9-induced mutations on *fw2.2* (Additional file 1: Table S7). The mutation type can be observed from the sequencing chromatograms (Fig. 5 and Additional file 2), when a heterozygous mutation (only one chromosome is mutated, while the other is not) or biallelic mutation (different mutations on two chromosomes) occurs in the target sequence, overlapping peaks appear after the target site, while the sequencing chromatogram only has a single peak in a homozygous mutation (two chromosomes have the same mutation) or non-mutant. The mutation sequences can be read directly from the sequencing files. Based on this, the mutation types were counted, and the editing efficiency were also counted following the formula: gene editing efficiency (%) = (number of gene-edited seedlings / number of resistant seedlings) × 100%.

### Quantitative real-time PCR (qRT-PCR) analysis of *fw2.2* in gene-edited seedlings

In order to study whether gene editing has an effect on the expression of *fw2.2*, the wild *Lycium ruthenicum* seedlings and the gene-edited seedlings including X9 (homozygous), X17 (heterozygous), X20 (biallelic) and X21 (heterozygous) were selected for quantitative real-time PCR analysis. Actin (HQ415754.1) was used as an internal control. The primers used for qRT-PCR were designed on NCBI including actin -F: TACGAGGGTTACGCTTTGCC, actin -R: TTCCCGTTCAGCAGTGGTTG, Fw2.2-F: TGCCACTGCTTTGATGACCC, Fw2.2-R: TATAACGCACCTCTACCCGC. Thermocycling conditions were 95 ℃ for 1 min, followed by 40 cycles of 95 ℃ for 10 s, 55 ℃ for 5 s, and 72 ℃ for 15 s. Relative gene expression data were analyzed using the 2^–ΔΔCT^ [41].

## Supplementary Information

**Additional file 1: Table S1.** Establishment of regeneration system of *Lycium ruthenicum*. **Table S2.** Target sequence selection of *Lycium ruthenicum* fw2.2. **Table S3.** Establishment of genetic transformation system of *Lycium ruthenicum*. **Table S4.** Specific primers for *fw2.2* gene amplification. **Table S5.** Single target editing primers *fw2.2*–1-sgRNA and *fw2.2*–2-sgRNA. **Table S6.** Primer sequence of pCAMBIA1300-fw2.2–1/2 vector construction. **Table S7**. Detection primers of different target sites.

**Additional file 2.** Sequencing map information of gene-edited seedlings.

## Data Availability

We provide supporting and necessary data for publication of the article. All supporting data is presented in the article and Additional files. The strains and plasmids associated with this work will be made physically available by the authors upon reasonable request.
