# Supplementary material for: A highly efficient regeneration, genetic transformation system and induction of targeted mutations using CRISPR/Cas9 in Lycium ruthenicum
Source: Plant Methods. 2021 Jul 3;17:71. doi: 10.1186/s13007-021-00774-x (PMC8254353; doi:10.1186/s13007-021-00774-x)

Additional file 2 Sequencing map information of gene-edited seedlings. X refers to pCAMBIA1300-f*w2. 2*-1/2 double target editing seedlings; Y refers to pCAMBIA1300-fw*2. 2*-1 single target editing seedlings; Z refers to pCAMBIA1300-*fw2. 2*-2 single target editing seedlings.

X1 *fw2.2*-1 biallelic mutation

A0: TCTTCCAGA TCATATTGCCCTCTCATT

A1: TCTTCCAGA T TCATATTGCCCTCTCATT +1

A2: TCTTCCAG---------ATATTGCCCTCTCATT -3

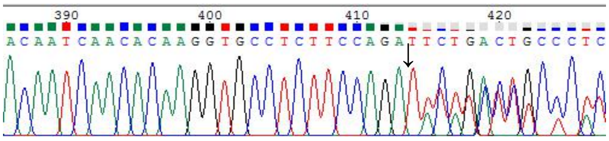

X5 *fw2.2*-1 biallelic mutation

A0: TCTTCCAGA TCATATTGCCCTCTCATT

A1: TCTTCCAGA T TCATATTGCCCTCTCATT +1

A2: TCTTCCAGA--------------TGCCCTCTCATT -6

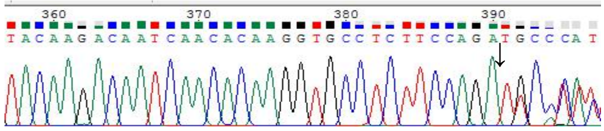

X7 *fw2.2*-2 heterozygous mutation

A0: CCTGCTAACT GTTTGGTTACTTGTGTTT

A1: CCTGCTAACT T GTTTGGTTACTTGTGTTT +1

A2: CCTGCTAACT GTTTGGTTACTTGTGTTT

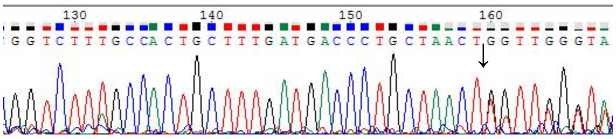

X9/X33 *fw2.2*-1 homozygous mutation

A0: AATGAGAGGGCAATATGA TCTGGAAGAG

A1: AATGAGAGGGCAATATGA**A** TCTGGAAGAG +1

A2: AATGAGAGGGCAATATGA**A** TCTGGAAGAG +1

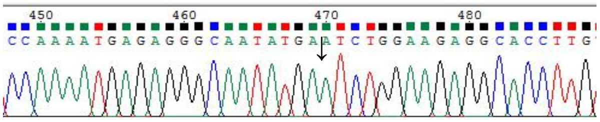

X18/X19 *fw2.2*-1 heterozygous mutation

A0: AAGGTGCCTCTTCCAGATCATATTGCCC

A1: AAGGTGCC-------------------------------TTTT -25

A2: AAGGTGCCTCTTCCAGATCATATTGCCC

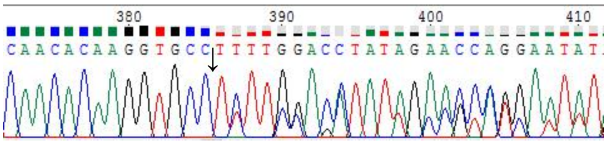

X30 *fw2.2*-1 biallelic mutation

A0: GGTGCCTCTTCCAGA TCATATTGCC

A1: GGTGCCTCTTCCAG---------ATATTGCC -3

A2: GGTGCCTCTTCCAGA C TCATATTGCC +1

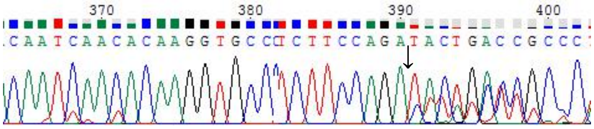

X37 *fw2.2*-1 biallelic mutation

A0: ACAAGGTGCCTCTTCCAGA TCATATTGC

A1: ACAAGGTGCCTCTTCCAGA T TCATATTGC +1

A2: ACAAGGTGCCTCTTCCAGA----------ATTGC -4

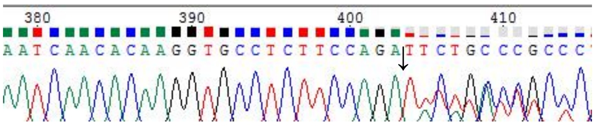

Y1 *fw2.2*-1 heterozygous mutation

A0: GGTGCCTCTTCCAGATCATATTGCC

A1: GGTGCCTCTTCCAGATC-------------C -7

A2: GGTGCCTCTTCCAGATCATATTGCC

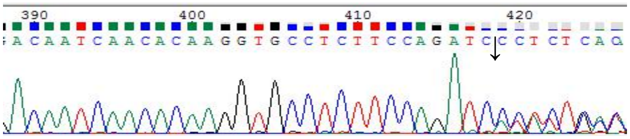

Y14 *fw2.2*-1 heterozygous mutation

A0: GGTGCCTCTTCCAGATCATATTGCC

A1: GGTGCCTCTTCCAG------ATATTGCC -3

A2: GGTGCCTCTTCCAGATCATATTGCC

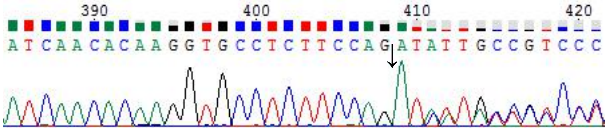

Z1/Z2 *fw2.2*-2 homozygous mutation

A0: CCTGCTAACTGTTTGGTTACTTGTGTTT

A1: CCTGCTAACT----------------------TGTGTTT -11

A2: CCTGCTAACT----------------------TGTGTTT -11

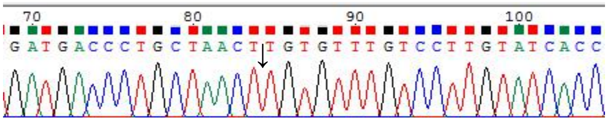

Z3 *fw2.2*-2 heterozygous mutation

A0: CCTGCTAACT GTTTGGTTACTTGTGTTT

A1: CCTGCTAACT T GTTTGGTTACTTGTGTTT +1

A2: CCTGCTAACT GTTTGGTTACTTGTGTTT

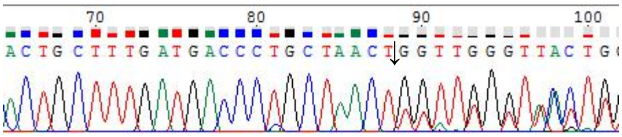

Z6 *fw2.2*-2 heterozygous mutation

A0: CCTGCTAACTGTTTGGTTACTTGTGTTT

A1: CCTGCTA------GTTTGGTTACTTGTGTTT -3

A2: CCTGCTAACTGTTTGGTTACTTGTGTTT

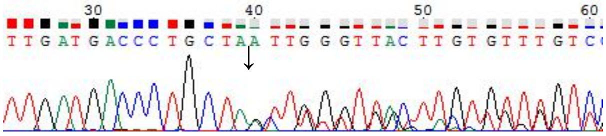

Z11 *fw2.2*-2 biallelic mutation

A0: GGACAAACACAAGTAACCAAAC AGTTAGCAGGG

A1: GGACAAACACAAGTAACCAAAC GAGTTAGCAGGG +1

A2: AAGG---------------------------------------AGGGTCATCAAA -27

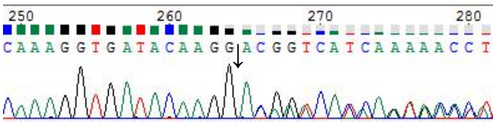

Supplement: Supplementary file 2 — Additional file 2. Sequencing map information of gene-edited seedlings. [file 13007_2021_774_MOESM2_ESM.pdf]
